# RESPONSE TO THE EDITORIAL “PSYCHO-EMOTIONAL CARE IN A NEONATAL UNIT DURING THE COVID-19 PANDEMIC”

**DOI:** 10.1590/1984-0462/2022/40/2020457

**Published:** 2021-06-25

**Authors:** Cristiane Ajnamei dos Santos Alfaya

**Affiliations:** a Health Sciences Center, Universidade Federal do Recôncavo da Bahia, Santo Antônio de Jesus, BA, Brazil.

Dear Authors,

The Editorial of Revista Paulista de Pediatria entitled “Psycho-emotional care in a neonatal unit during the COVID-19 pandemic” contributes to a deep reflection on maternal and child mental health. Based on Winnicott’s theoretical concepts, which are described in this text, the important discussion on the “caring” topic continues, especially in the current context of the COVID-19 pandemic.^1^

Overall, the act of caring brings with it a burden of responsibility that can lead to feelings and experiences of physical, psychological, social, and institutional exhaustion. In institutional spaces where health is taken care in order to promote well-being and healing, especially of the baby in a neonatal unit, it is necessary to consider both the baby’s period of development and the state of emotional availability that the healthcare team can offer to promote the socio-affective development of children and their family.

From the perspective of the initial maturational processes, according to Winnicott’s psychoanalytic theory, the individual’s mental health comprises three main aspects: the state of “going-on-being” that, according to the author, has a continuous potential; the sense of “feeling real,” that is, one’s feeling of existing and living in the body itself; and one’s sense of genuinely “doing,” through creativity and with autonomy. In order for these processes to achieve the development of the true self, that is, the “being” and the “doing” with creativity and autonomy, Winnicott postulated that, during the last months of pregnancy and the first weeks after childbirth, the mother experiences a special psychological state called “primary maternal preoccupation.” This state of increased sensitivity, which women experience in the pregnancy-puerperium period, allows the mother to meet the baby’s needs through projective identification. Thus, the mother’s past experiences as a daughter, her expectations as the baby’s parent, as well as the mother’s expectations and experiences with her own mother (the baby’s grandmother), can be observed in the mother’s interaction with the baby in the present. Therefore, the current mother-baby interaction involves the objective and subjective aspects of past and present interactions (expectations and experiences) in addition to the different contexts and current social conditions in which the mother-baby dyad is inserted. During the pregnancy-puerperium period, according to Winnicott, women experience a partial regression of the ego in order to identify with the baby and meet the child’s needs. Hence, it is paramount that the mother experiences a good enough environment (“holding”) in the present, in such a way to perform the function of caring for her baby with the predominance of current and positive subjective experiences.^2^ ^,^ ^3^

In the current context of the COVID-19 pandemic, the neonatal intensive care unit, together with healthcare professionals, has been performing the aforementioned functions, which are deemed essential to promote the baby’s physical and psychological maturational processes. Winnicott highlights the maturational processes as linked to the concept of *holding*, which involves the experience of physical protection due to the baby’s skin sensitivity – touch, temperature – as well as its auditory, visual, and touch sensitivity. This is also because the baby is still unaware of its full existence (fragments of the ego or subjective experiences) as well as everything other than itself (external environment). In this sense, the aim of the initial period of human development is the integration of subjective experiences (pulsion, instincts, perceptual and motor capacities) to form the core of the self (ego) and personalization – to acquire the sense that the body houses the true self. Moreover, according to this theorist, holding involves the routine of physical care for the baby throughout the day and night as well as expressing love and affection with the physical contact of holding the child in one’s arms. The environment provided by holding (mother/parents, primary caregiver, or healthcare professional) works as an auxiliary ego, being a determining factor in the transition from the state of non-integration (baby’s absolute dependence) to that of integration (from relative dependence to independence). These states can be later observed in children’s exploratory behavior, in their creativity, autonomy, initiative, attention, among others, during the act of playing. The individuals’ continuous process of going-on-being through subjective experiences in the interaction with the environment (mother/parents, primary caregiver, or healthcare professional) will form the basis for the development of the individuals’ healthy potential toward autonomy and independence such as senses of trust, belonging, self-esteem, safety, emotional regulation, among others.^4^

Winnicott highlights the processes of emotional maturation as being innate to the individual toward life, that is, to the encounter of internal (I) and external (other) objects. The baby’s opportunity to find these objects, due to the subjective experiences resulting from interactions with the environment, allows individuals to construct senses and meanings about themselves and the other (world). Therefore, holding (good enough environment), which reflects the image of oneself and that of the other, initially merged and undifferentiated, allows for the separation and true differentiation of real objects through interactive exchanges and subjective experiences of the self.^2^ ^,^ ^3^ ^,^ ^4^

Integration is achieved by two types of experiences: on the one hand, the mother’s support, who “collects the little fragments of the ego,” is especially important, enabling the child to feel integrated with her; on the other hand, there is a type of experience that tends to bring personality together in an internal base (the baby’s mental activity). There comes a time when children, thanks to the aforementioned experiences, manage to bring together the cores of their ego, having the notion that they are different from the world surrounding them. This moment of differentiation between “me” and “not me” can be dangerous for the baby, as the outside can be perceived as agonizing and threatening. These threats are neutralized, in a healthy development, by the existence of loving care on the part of the mother. The true self begins to have life through the strength given to the infant’s weak ego by the mother, when implementing the child’s omnipotence expressions. Again, the role of the environment (mother/parents, primary caregiver, healthcare professional) is to provide the baby with an auxiliary ego that allows it to integrate its bodily sensations, environmental stimuli, and motor skills. Otherwise, babies may replace the protection they lacks with one “made” by them, wrapping themselves in a shell at the expense of which the false self grows and develops. Individuals develop as an extension of the shell, an extension of the environment that is not good enough, threatening and hostile, which has failed to interpret their needs, being unable to implement the infant omnipotence and imposing the infant gesture. The false self, especially when it is at the most pathological extreme of the scale, is usually accompanied by a subjective feeling of emptiness, futility, and unreality.^5^

Winnicott defines personalization as “someone’s feeling of being in their own body.” The author suggests that normal development would lead individuals to achieve a body scheme, a psyche-soma unit, as called by Winnicott, which forms their body scheme as a whole – it is interpenetrated and developed in a dialectical relationship and presents the diversity paradox in the unity. As development progresses, children have a relatively integrated ego and the feeling that their own core inhabits their body. Children and the world are two separate things. The next step is to achieve the adaptation to reality. At this stage, the parents, primary caregiver, healthcare professionals have the function of providing children with the elements of reality with which they will be able to create their psychic image and that of the outside world.^5^

In this sense, intervention strategies in the routine of care in neonatal units aimed at the newborn, at the family, as well as those aimed at the caregiver described by the authors Denise Streit Morsch, Zaira Aparecida de Oliveira Custódio, and Zeni Carvalho Lamy in the Editorial of Revista Paulista de Pediatria, are essential for promoting physical, psychological, social, and institutional health in the bidirectional relationship established between families with babies in intensive care units and healthcare professionals working in such units.
